# HSPA12A Stimulates p38/ERK-AP-1 Signaling to Promote Angiogenesis and Is Required for Functional Recovery Postmyocardial Infarction

**DOI:** 10.1155/2022/2333848

**Published:** 2022-06-22

**Authors:** Tingting Li, Jun Wu, Wansu Yu, Qian Mao, Hao Cheng, Xiaojin Zhang, Yuehua Li, Chuanfu Li, Zhengnian Ding, Li Liu

**Affiliations:** ^1^Department of Geriatrics, The First Affiliated Hospital of Nanjing Medical University, Nanjing, China; ^2^Department of Anesthesiology, First Affiliated Hospital of Nanjing Medical University, Nanjing, China; ^3^Department of Anesthesiology, The First Affiliated Hospital with Wannan Medical College, Wuhu, China; ^4^Key Laboratory of Targeted Intervention of Cardiovascular Disease, Collaborative Innovation Center for Cardiovascular Disease Translational Medicine, Nanjing Medical University, Nanjing, China; ^5^Department of Surgery, East Tennessee State University, Johnson City, TN, USA

## Abstract

Angiogenesis plays a critical role in wound healing postmyocardial infarction (MI). However, there is still a lack of ideal angiogenic therapeutics for rescuing ischemic hearts clinically, suggesting that a more understanding regarding angiogenesis regulation is urgently needed. Heat shock protein A12A (HSPA12A) is an atypical member of the HSP70 family. Here, we demonstrated that HSPA12A was upregulated during endothelial tube formation, a characteristic of *in vitro* angiogenesis. Intriguingly, overexpression of HSPA12A promoted *in vitro* angiogenic characteristics including proliferation, migration, and tube formation of endothelial cells. By contrast, deficiency of HSPA12A impaired myocardial angiogenesis and worsened cardiac dysfunction post-MI in mice. The expression of genes related to angiogenesis (VEGF, VEGFR2, and Ang-1) was decreased by HSPA12A deficiency in MI hearts of mice, whereas their expression was increased by HSPA12A overexpression in endothelial cells. HSPA12A overexpression in endothelial cells increased phosphorylation levels and nuclear localization of AP-1, a transcription factor dominating angiogenic gene expression. Also, HSPA12A increased p38 and ERK phosphorylation levels, whereas inhibition of p38 or ERKs diminished the HSPA12A-promoted AP-1 phosphorylation and nuclear localization, as well as VEGF and VEGFR2 expression in endothelial cells. Notably, inhibition of either p38 or ERKs diminished the HSPA12A-promoted *in vitro* angiogenesis characteristics. The findings identified HSPA12A as a novel angiogenesis activator, and HSPA12A might represent a viable strategy for the management of myocardial healing in patients with ischemic heart diseases.

## 1. Introduction

Myocardial infarction (MI) is the irreversible death of cardiac cells secondary to prolonged ischemia and can transit to heart failure which accounts for one of the leading causes of death worldwide [1, 2]. Angiogenesis, the new blood vessel formation, has been shown to have the potential to rescue ischemic myocardia early after MI and also is critical to prevent long-term cardiac remodeling to prevent heart failure development [3–5]. Therefore, managing angiogenesis to timely and effectively restore blood flow is a promising therapeutic approach for healing MI hearts.

Angiogenesis is a complex process initiated by the proliferation and migration of endothelial cells (ECs) in response to angiogenic factors. Among these angiogenic factors, the vascular endothelial growth factor (VEGF) is considered especially critical due to its powerful role in the activation of EC proliferation and migration by binding to its receptors such as VEGFR2 [6, 7]. VEGF is also helpful for EC survival under ischemic conditions. In addition to the VEGF-dependent pathway, the Angiopoietin- (Ang-) mediated signaling is necessary for angiogenesis through promoting EC proliferation and migration, maturing new blood vessels, and maintaining vessel stability [8, 9]. Accordingly, inhibitors targeting VEGF- or Ang-dependent pathways suppress solid tumor growth clinically or preclinically and also are effective for age-related macular degeneration treatment [10, 11]. Specifically, several clinical trials for evaluating therapeutic angiogenesis after MI have been performed. For example, EPICCURE is an ongoing clinical trial by delivering VEGF mRNA to ischemic but viable myocardia to assay its safety and potential angiogenic effects [12]. However, a more comprehensive understanding regarding angiogenesis regulation is still needed.

Activator protein-1 (AP-1) is a protein dimer formed by Jun, Fos, or ATF and emerged as a critical transcription factor for angiogenesis at both the basal and inducible levels through controlling VEGF and other angiogenic gene expressions [13]. Following phosphorylated by kinases such as MAPK (ERKs, p38, and JNKs) and PI3K/Akt signaling, AP-1 translocates to nuclei to drive target gene expression [14, 15]. Therefore, activating AP-1-mediated angiogenic signaling might be beneficial for MI repairment.

Heat shock protein A12A (HSPA12A) was identified in 2003 and classed as a distant member of the heat shock protein 70 family [16]. Subsequent studies revealed the downregulation of HSPA12A in the brains of patients with schizophrenia [17]. Recently, we have reported that HSPA12A is necessary for cerebral protection from stroke, for development of obesity and nonalcohol liver disease, and for growth of liver cancer [18–21]. Particularly, we found that HSPA12A facilitates angiogenesis in mouse liver carcinoma [21] and increases basal levels of ERK phosphorylation and VEGF expression in endothelial cells [21, 22]. Therefore, it is possible that HSPA12A might promote angiogenesis to attenuate post-MI cardiac dysfunction.

To test this possibility, we examined the effects of HSPA12A on angiogenesis and post-MI cardiac performance. We found that HSPA12A was upregulated during endothelial tube formation, while the knockout of HSPA12A in mice impaired cardiac angiogenesis and worsened cardiac dysfunction post-MI. Further molecular analysis revealed that the HSPA12A-promoted angiogenesis was mediated by activating the p38/ERK-AP-1 signaling axis. The findings indicate that HSPA12A may represent a potential target for cardioprotection against ischemia through promoting angiogenesis.

## 2. Materials and Methods

### 2.1. Reagents and Antibodies

The MTT (3-(4,5-dimethylthiazol-2-yl)-2,5-diphenyltetrazolium bromide) reagent and primary antibodies for *α*-smooth muscle actin (*α*-SMA) and *α*-tubulin were purchased from Sigma-Aldrich (St. Louis, MO). The primary antibody for GAPDH was from Bioworld Technology (St. Louis Park, MN). Primary antibodies for c-Jun, phosphor-c-Jun (p-c-Jun), p38, phosphor-p38, ERKs, phosphor-ERKs (p-ERKs), JNKs, and phosphor-JNKs (p-JNKs) were purchased from Cell Signaling Technology (Beverly, MA). The primary antibody for Ang-1 was from Santa-Cruz Biotechnology (Dallas, TX). The primary antibody for VEGFR2 was from SAB (Baltimore, MD). Primary antibodies for HSPA12A and CD31 were from Abcam (Cambridge, MA). The primary antibody for VEGF was purchased from Millipore (Billerica, MA). Matrigel was a product from BD Biosciences (San Jose, CA). Bovine serum albumin (BSA) was a product from Roche (Basel, Switzerland). Normal goat serum was a product of Jackson Immuno Research (West Grove, PA). Fetal bovine serum (FBS) and the M199 medium were products from Biological Industries (Kibbutz Beit HaEmek, Israel). PD98059 and SB203580 were from MedChemExpress (Monmouth Junction, NJ).

### 2.2. Animals

Conditional *Hspa12a* knockout mice (*Hspa12a*^−/−^) were generated using the *lox*P and *Cre* recombinant system as described in our previous studies [19, 20, 23]. Briefly, the region of the *Hspa12a* gene containing exons 2–4 was retrieved from a 129/sv BAC clone (BAC/PAC Resources Center, Oakland, CA) using a retrieval vector containing two homologous arms. Exons 2 and 3 were replaced by *lox*P sites flanking a PGK-neo cassette as a positive selection marker. Embryonic stem cells were electroporated with the linearized targeting vector, selected, and then expanded for Southern blotting analysis. Chimeric mice (*Hspa12a*^*flox*/*+*^) were generated by injecting embryonic stem cells into C57BL/6 blastocysts, followed by transfer into pseudopregnant mice. To remove the *Hspa12a* gene, the chimeric mice were crossed with EIIa-Cre transgenic mice. The mice were bred at the Model Animal Research Center of Nanjing University and were maintained in the Animal Laboratory Resource Facility of the same institution. The animal care and experimental protocols were approved by Nanjing University's Committee on Animal Care. All experiments conformed to the *Guide for the Care and Use of Laboratory Animals* published by the US National Institutes of Health (NIH Publication, 8th Edition, 2011).

Mice (C57BL/6 strain) were randomly assigned to all analyses. Investigators were blinded to the histological analysis. Investigators involved in animal handling, sampling, and raw data collection were not blinded.

### 2.3. Myocardial Infarction (MI) Surgery in Mice

MI was induced in 8- to 12-week-old male *Hspa12a*^−/−^ mice and their wild-type (WT) littermates by permanently ligating the left anterior descending coronary (LAD) according to our previous methods [2, 3]. Briefly, mice were anesthetized by inhalation of 1.5–2% isoflurane. The adequacy of anesthesia was assayed by the disappearance of righting reflex and pedal withdrawal reflex. After anesthesia, mice were subjected to mechanical ventilation, chest opening along the left sternal border, and permanent LAD ligation. For sham operation, the same surgery was conducted except for the LAD occlusion. Body temperature was maintained at 36.8–37.1°C using a heating platform throughout the surgical procedure. For tissue collection, mice were sacrificed by overdose anesthesia (pentobarbital sodium 150 mg/kg intraperitoneal injection) and cervical dislocation.

### 2.4. Echocardiographic Measurement in Mice

Two-dimensional echocardiography was performed to examine cardiac function using the Vevo770 system equipped with a 35 MHz transducer (VisualSonics, Toronto, Canada) according to our previous methods [2, 3, 24]. Briefly, after being anesthetized by inhalation with 1.2% isoflurane, mice were laid on the equipped plate and chest hair was shaved. Subsequently, the measurements were performed by an observer blinded to the treatment. The parameters were obtained in the M-mode tracings at the papillary muscle level and averaged using three to five cardiac cycles. Ejection fraction (EF%) and fraction shortening (FS%) of left ventricles were used to indicate cardiac systolic function.

### 2.5. Primary Human Umbilical Vein Endothelial Cell (HUVEC) Isolation and Growth

HUVECs were obtained from the umbilical vein cords of normal pregnancies according to our previous methods [22, 25]. Briefly, endothelial cells were dissociated from umbilical veins with 0.25% trypsin and grown in the M199 medium supplemented with 10% FBS and 0.5 ng/ml bFGF. The HUVECs in passages 2 to 5 were used in the experiments. The studies were approved by the Ethical Board of the First Affiliated Hospital of Nanjing Medical University (#2021-SR-104). All the human study procedures were followed in accordance with the ethical standards of the responsible committee on human experimentation and with the Helsinki Declaration of 1975, as revised in 2000. Human pulmonary artery endothelial cells (HPAEC) were obtained from Sciencell Research Laboratories (Carlsbad, CA, USA) and grown in endothelial cell medium-2 (ECM-2) (Sciencell) with 5% FBS and 1% endothelial cell growth factors according to previous methods [26].

### 2.6. Overexpression of HSPA12A in HUVECs

To overexpress HSPA12A (*Hspa12a*^*o*/*e*^) in HUVECs, cells were infected with adenovirus that carrying the *Hspa12a* expression coding sequence as our previous methods [19, 20, 23]. The adenoviral vector containing the 3 Flag-tagged mouse *Hspa12a* coding region (NM_175199) was generated by GeneChem Company (Shanghai, China). The scheme of virus construction is shown in Figure S1. The cells infected with empty adenovirus served as normal controls (NC).

For p38 or ERK inhibition, SB203580 (20 *μ*M) or PD98059 (25 *μ*M) were introduced to cell cultures 24 h after HSPA12A overexpression.

### 2.7. Matrigel-Based *In Vitro* Angiogenesis Assay

*In vitro* endothelial angiogenesis was assessed by tube formation using Matrigel-based assay [27]. Briefly, after HSPA12A overexpression for 24 h, HUVECs (1.5 × 10^4^ cells/well) were seeded on growth factor-reduced Matrigel-coated 96-well plates. Cells were photographed at 2.5 and 4 h after being grown on Matrigel using a microscope at a magnification of 40x. Tube formation was expressed as total branch length/field and master junctions/field using NIH ImageJ software.

### 2.8. Endothelial Cell Migration Assay

The migration capacity of HUVECs was measured by the scratch (or wound healing) assay according to our previous methods [28]. Briefly, HUVECs grown in 6-well plates were scratched with 200 *μ*l tips when cells reached 80% confluence. After scratching, cells were incubated with a 3% FBS-supplemented M199 medium and photographed at the indicated time points. The percentage of wound closure was analyzed by an image analyzer (NIH ImageJ software).

### 2.9. MTT Assay

MTT assays were performed to evaluate the viability of HUVECs after overexpression of HSPA12A for 24, 48, and 72 h according to our previous methods [21]. In brief, HUVECs were incubated with MTT (0.5 mg/ml) for 4 h. The crystals that formed were solubilized in DMSO, and the color was read on a Synergy HT plate reader at 570 nm (Synergy HT, BioTek, USA).

### 2.10. Western Blotting Analysis

Cellular proteins were prepared from infarcts of mice 7 days after MI or from HUVECs 24 h after HSPA12A overexpression [18]. Briefly, after separating by SDS-PAGE and transferring onto Immobilon-P membranes, the membranes were incubated with the primary antibodies and subsequently incubated with peroxidase-conjugated secondary antibodies. After detection with a chemiluminescent substrate, the signals were captured using scanning densitometry. Results from each set of experiments were presented as the relative integrated intensities (compared to those of controls).

### 2.11. Immunofluorescence Staining

For analyzing myocardial angiogenesis, cardiac tissues were collected transversely at papillary muscles for immunofluorescence staining according to our previous methods [2, 3, 29]. Briefly, the tissues were prepared for cryosectioning at a thickness of 4 *μ*m. After blocking with 7.5% normal goat serum (1 hour), tissue sections were probed with the primary antibody (4°C, overnight) and followed by incubation with secondary antibodies to visualize the staining. The DAPI reagent was used to counterstain nuclei. The staining was observed, captured, and quantified using a fluorescence microscope (Olympus, Japan).

For immunocytochemistry in primary endothelial cells, cells grown on coverslips were fixed with acetone/methanol (1 : 1) and followed by immunostaining as mentioned above.

### 2.12. EdU Incorporation Assay

EdU incorporation assay was performed to indicate cell proliferation according to our previous methods [21]. After overexpression of HSPA12A for 48 h, HUVECs were incubated with EdU (50 *μ*M, 2 h, 37°C). After fixation with 4% formaldehyde (30 min), cells were permeabilized with 0.5% Triton X-100 (room temperature, 10 min), followed by incubation with Apollo reaction cocktail (30 min). DAPI was used to counterstain nuclei. The proportion of cells that incorporated EdU was determined by fluorescence microscopy.

### 2.13. Histological Examination

Following MI for 14 days, cardiac tissues at papillary muscle levels were collected and fixed with 4% paraformaldehyde for 24 h. After paraffin-embedded sectioning was prepared, hematoxylin-eosin (HE) staining was performed. The staining was observed under a microscope.

### 2.14. Statistics

Results are presented as the mean ± standard deviation. Results were analyzed by Student's two-tailed unpaired *t*-test, one-way analysis of variance (ANOVA), or two-way analysis of variance, followed by a Tukey test as a post hoc test. *P* < 0.05 was considered statistically significant.

## 3. Results

### 3.1. HSPA12A Is Upregulated during Endothelial Tube Formation

**Figure 1:**
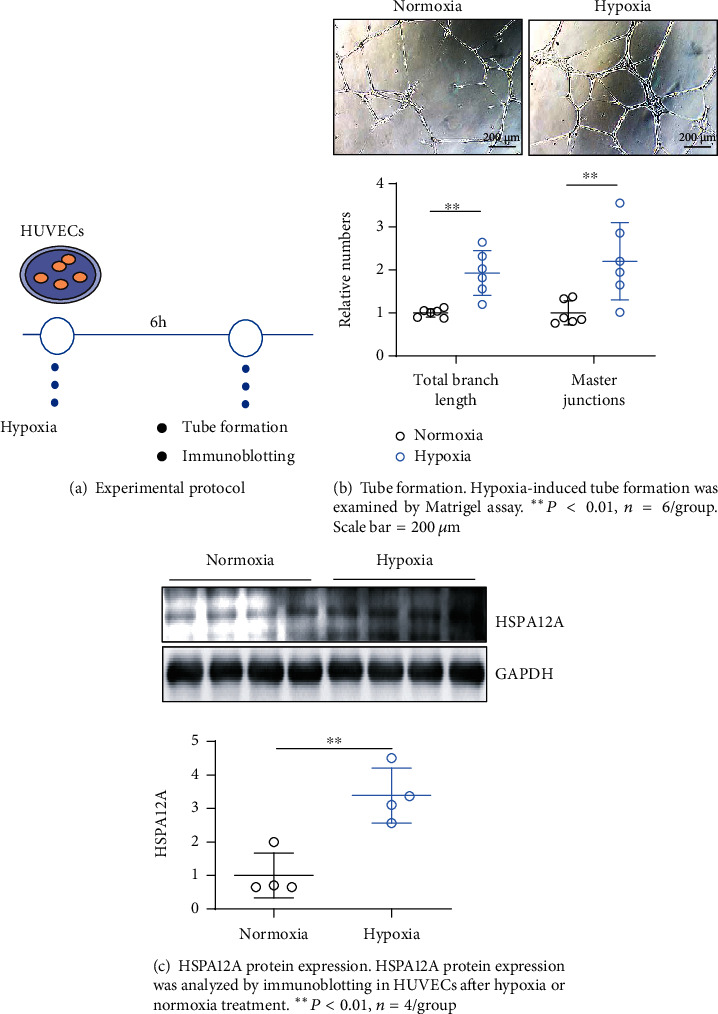
HSPA12A was upregulated during endothelial tube formation.

To investigate whether HSPA12A is involved in angiogenesis, an *in vitro* experiment was performed to examine HSPA12A expression in endothelial cells during tube formation. To this end, HUVECs grown on Matrigel-coated plates were subjected to hypoxia to induce tube formation according to previous studies [30]. Hypoxia was achieved by incubation of HUVECs with a medium containing 3% FBS in an incubator containing 1% O_2_, 94% N_2_, and 5% CO_2_ for 6 h (Figure 1(a)). We found that hypoxia induced tube formation, as indicated by increases of both total branch length and master junction numbers (Figure 1(b)). Notably, HSPA12A displayed a 2-fold higher expression during hypoxia-induced tube formation than normoxia HUVECs (Figure 1(c)).

### 3.2. HSPA12A Promotes Proliferation of HUVECs

**Figure 2:**
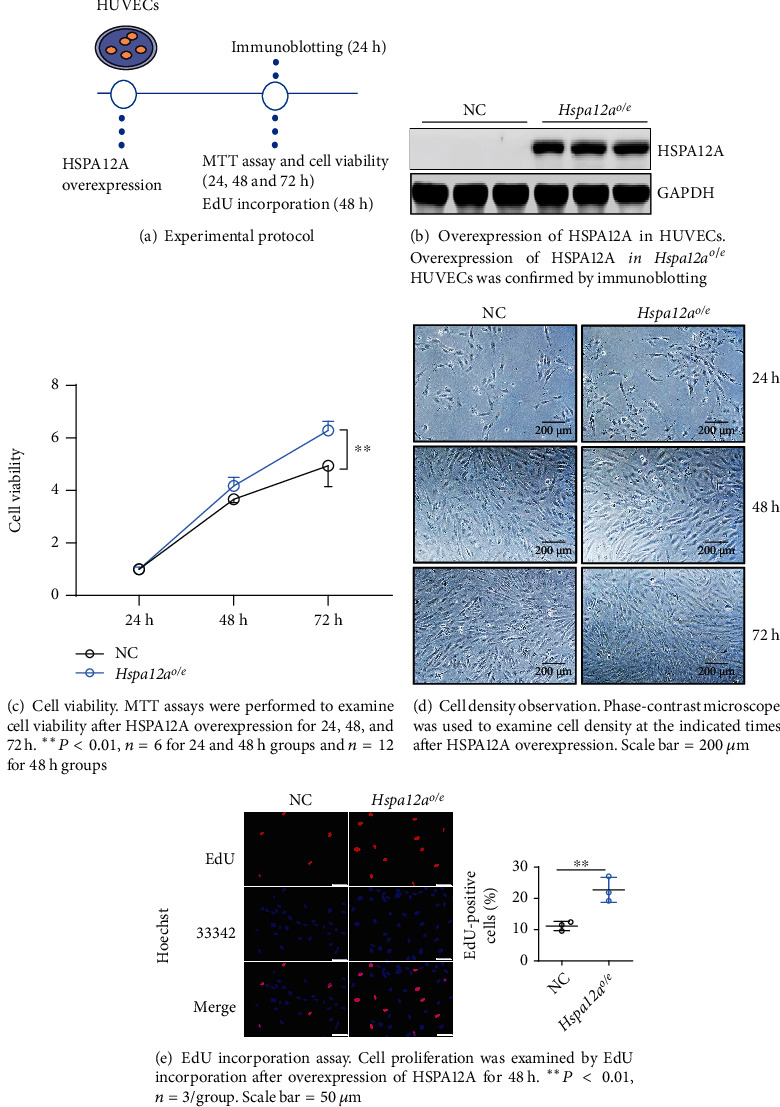
HSPA12A promoted proliferation of HUVECs.

The upregulation of HSPA12A in endothelial cells during tube formation prompts us to elucidate whether HSPA12A plays a role in angiogenesis. To this end, HSPA12A was overexpressed (*Hspa12a*^*o*/*e*^) in HUVECs by infection with *Hspa12a*-adenovirus, and cells infected with empty adenovirus served as normal controls (NC) (Figures 2(a) and 2(b), Figure S2). Endothelial proliferation is a key step for angiogenesis [31]. MTT assay showed higher viability in *Hspa12a*^*o*/*e*^ HUVECs after HSPA12A overexpression for 72 h than the time-matched NC group (Figure 2(c)). This finding was confirmed by cellular morphological examination (Figure 2(d)). Moreover, *Hspa12a*^*o*/*e*^ HUVECs displayed more EdU incorporation than NC (Figure 2(e)). Together, the data indicate that endothelial proliferation is promoted by HSPA12A.

### 3.3. HSPA12A Promotes Motility and Tube Formation of HUVECs

**Figure 3:**
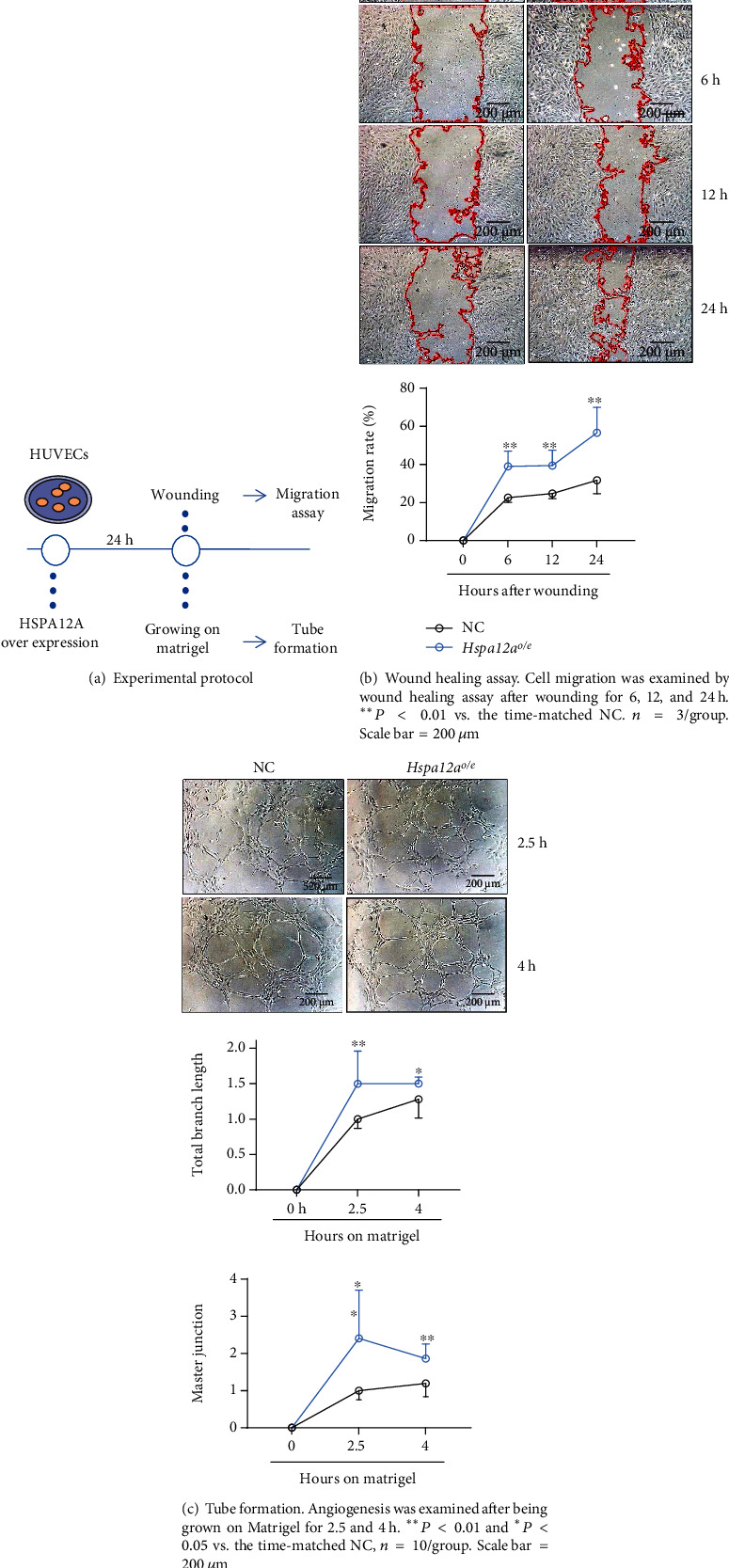
HSPA12A promoted motility and tube formation of HUVECs.

Motility and tube formation of endothelial cells are essential for angiogenesis. To examine the effect of HSPA12A on endothelial motile capacity, wound healing assay was performed as we described recently [28] (Figure 3(a)). We found that overexpression of HSPA12A in HUVECs promoted the migration rate after wounding for 6, 12, and 24 h, respectively, compared to the time-matched NC group (Figure 3(b)). Moreover, overexpression of HSPA12A in HPAEC promoted the migration capacity in the wound healing assay (Figure S3). Next, tube formation was examined using Matrigel-based assay [27]. *Hspa12a*^*o*/*e*^ HUVECs displayed significantly longer branch length after growing on Matrigel for 2.5 and 4 h than the time-matched NC group (Figure 3(c)). Similarly, significantly more master junctions were demonstrated in *Hspa12a*^*o*/*e*^ HUVECs after growing on Matrigel than in the time-matched NC group (Figure 3(c)).

### 3.4. HSPA12A Upregulates Angiogenic Gene Expression in HUVECs

**Figure 4:**
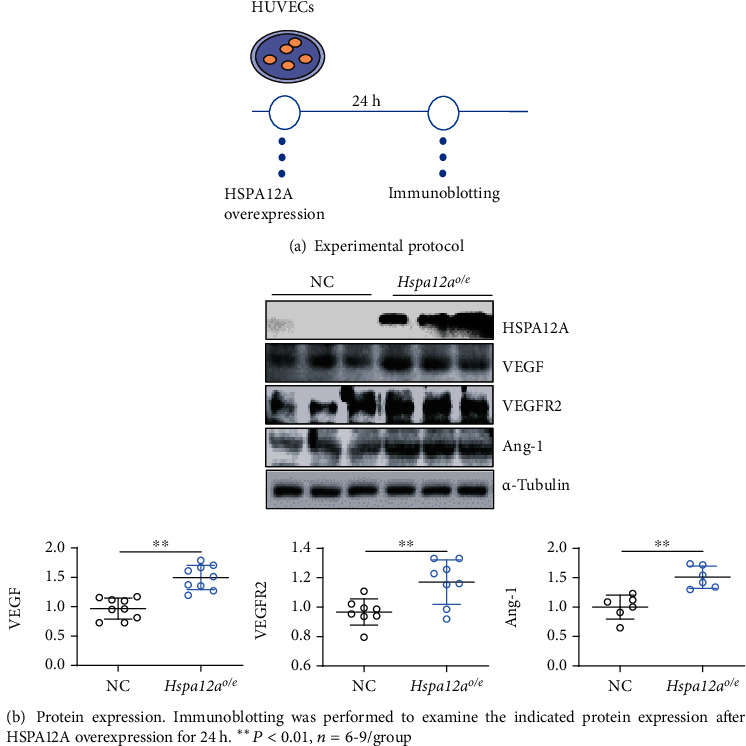
HSPA12A upregulated expression of angiogenic genes of HUVECs.

Angiogenesis is strongly regulated by VEGF signaling through VEGF receptors such as VFGFR2 [32]. Overexpression of HSPA12A in HUVECs for 24 h significantly increased VEGF expression compared to the NC group (Figures 4(a) and 4(b)). Similar results were found in VEGFR2 examination, which showed markedly higher expression in *Hspa12a*^*o*/*e*^ HUVECs than in the NC group (Figure 4(b)). Angiopoietin-1 (Ang-1) is also important for angiogenesis through promoting endothelial proliferation and migration, maturing new blood vessels, and maintaining vessel stability [33–35]. Intriguingly, significantly higher Ang-1 protein expression was found in *Hspa12a*^*o*/*e*^ HUVECs compared to the NC group (Figure 4(b)).

### 3.5. HSPA12A Increases c-Jun/AP-1 Phosphorylation and Nuclear Localization in HUVECs

**Figure 5:**
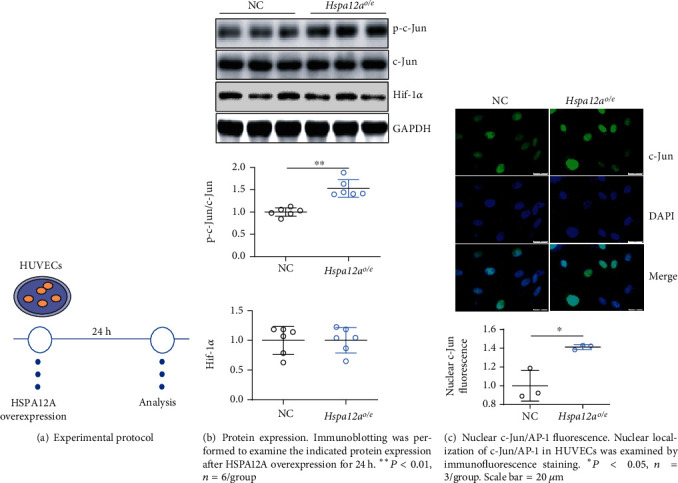
HSPA12A increased c-Jun/AP-1 phosphorylation and nuclear localization in HUVECs.

The expression of angiogenic genes, such as VEGF, is regulated by several transcriptional factors including AP-1 and Hif-1*α* [36, 37]. No difference in Hif-1*α* expression levels was found between the *Hspa12a*^*o*/*e*^ and NC HUVEC groups (Figures 5(a) and 5(b)). However, c-Jun, a component of AP-1 dimers, displayed significantly higher phosphorylation levels in *Hspa12a*^*o*/*e*^ HUVECs than in the NC group (Figure 5(b)). Accordingly, more nuclear content of c-Jun/AP-1 was found in *Hspa12a*^*o*/*e*^ HUVECs than in NC (Figure 5(c)). Moreover, in the Ad-HSPA12A group, the HUVECs with stronger HSPA12A staining also displayed stronger c-Jun staining in nuclei (Figure S4).

### 3.6. HSPA12A Increases p38 and ERK Phosphorylation Levels

**Figure 6:**
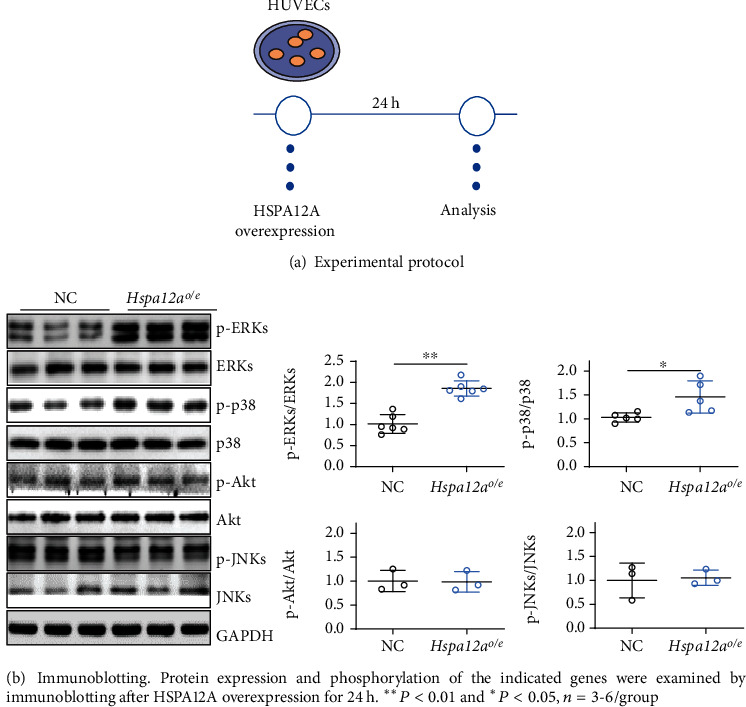
HSPA12A increased p38 and ERK phosphorylation levels in HUVECs.

The phosphorylation of c-Jun/AP-1 is critical for its translocation to nuclei, where it exerts a transcription role to drive target gene expression. Considering that c-Jun can be phosphorylated by MAPKs and Akt [14, 15, 38], we examined the effects of HSPA12A on phosphorylation levels of MAPKs (p38, ERKs, and JNKs) and Akt (Figure 6(a)). No difference in phosphorylation levels of JNKs and Akt was found between *Hspa12a*^*o*/*e*^ and NC (Figure 6(b)). However, *Hspa12a*^*o*/*e*^ HUVECs displayed significantly higher phosphorylation levels of p38 and ERKs than NC (Figure 6(b)).

### 3.7. Inhibition of p38 Attenuated the HSPA12A-Promoted c-Jun/AP-1 Phosphorylation, Angiogenic Gene Expression, and *In Vitro* Angiogenic Phenotypes of HUVECs

**Figure 7:**
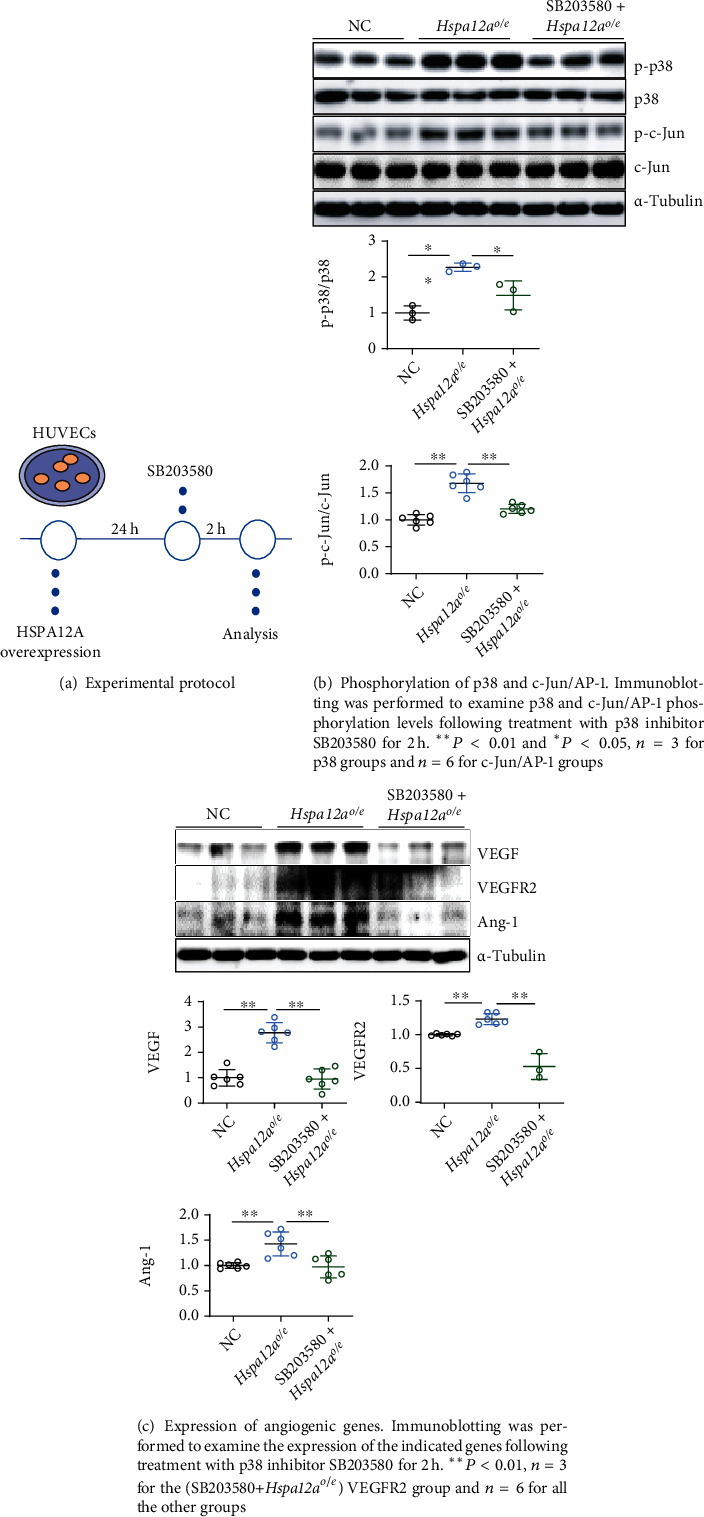
Inhibition of p38 reversed the HSPA12A-promoted c-Jun/AP-1 phosphorylation and angiogenic gene expression of HUVECs.

**Figure 8:**
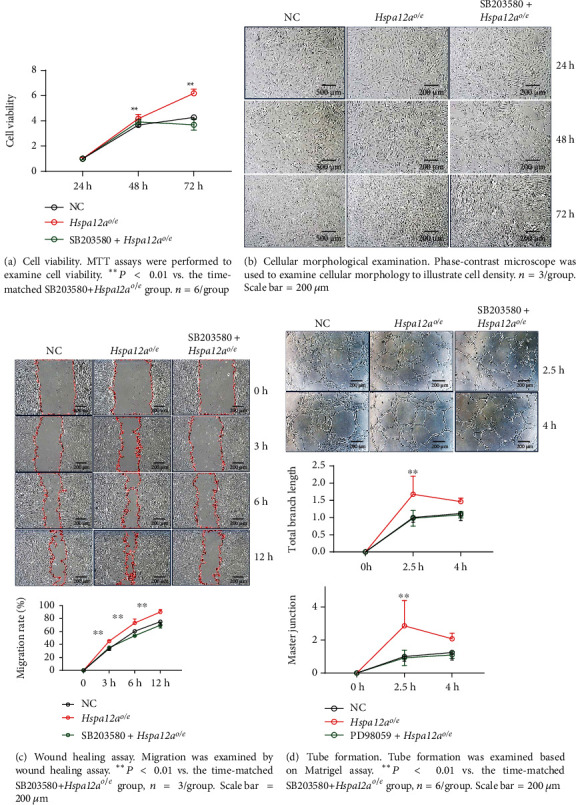
Inhibition of p38 abolished the HSPA12A-promoted *in vitro* angiogenic phenotypes of HUVECs.

To determine the roles of p38 activation in HSPA12A-promoted *in vitro* angiogenesis, HUVECs were treated with p38 inhibitor SB203580 for 2 h (Figure 7(a)). Intriguingly, SB203580 removed the HSPA12A-induced increase of c-Jun/AP-1 phosphorylation (Figure 7(b)). The upregulation of VEGF and VEGFR2 by HSPA12A overexpression was also abolished by SB203580 (Figure 7(c)). Notably, HSPA12A-induced promotion of proliferation of HUVECs was diminished by SB203580, as evidenced by both MTT and morphological examination (Figures 8(a) and 8(b)). Moreover, HSPA12A-induced promotion of migration ability and tube formation capacity was removed by SB203580 (Figures 8(c) and 8(d)), suggesting that the HSPA12A-promoted angiogenesis is p38 dependent.

**Figure 9:**
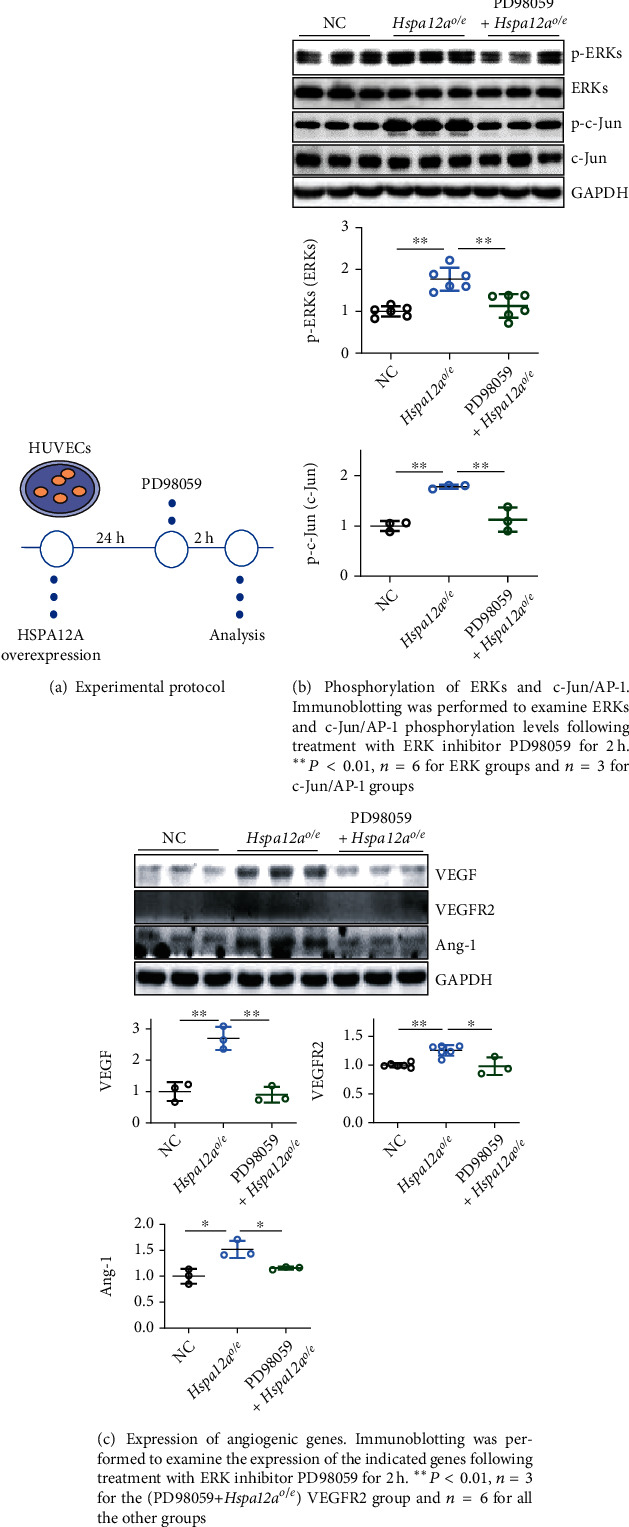
Inhibition of ERKs reversed the HSPA12A-promoted c-Jun/AP-1 phosphorylation and angiogenic gene expression of HUVECs.

**Figure 10:**
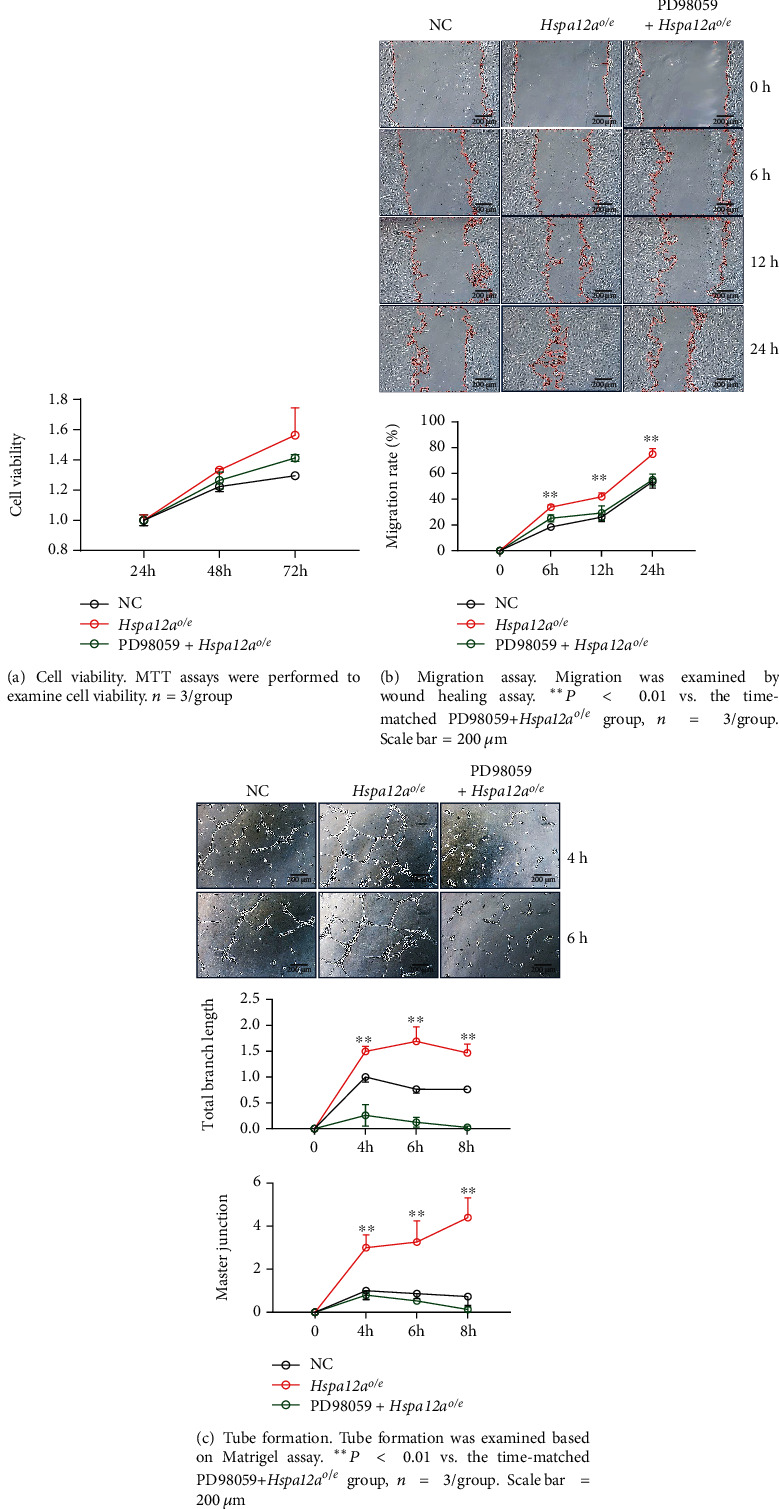
Inhibition of p-ERKs attenuated the HSPA12A-promoted *in vitro* angiogenic phenotypes of HUVECs.

Considering that ERK phosphorylation was also increased by HSPA12A, we therefore investigated the roles of ERKs in HSPA12A-promoted angiogenesis. To this end, HUVECs were treated with ERK inhibitor PD98059 (Figure 9(a)). ERK inhibition with PD98059 attenuated the HSPA12A-induced increases of c-Jun/AP-1 phosphorylation of VEGF and VEGFR2 expression (Figures 9(b) and 9(c)). Though inhibition of ERKs did not impact the HSPA12A-promoted cell proliferation, the HSPA12A-promoted migration and tube formation were attenuated following PD98059 treatment (Figures 10(a)–10(c)).

### 3.8. Deficiency of HSPA12A in Mice Impaired Angiogenesis and Worsened Cardiac Dysfunction after MI in Mice

**Figure 11:**
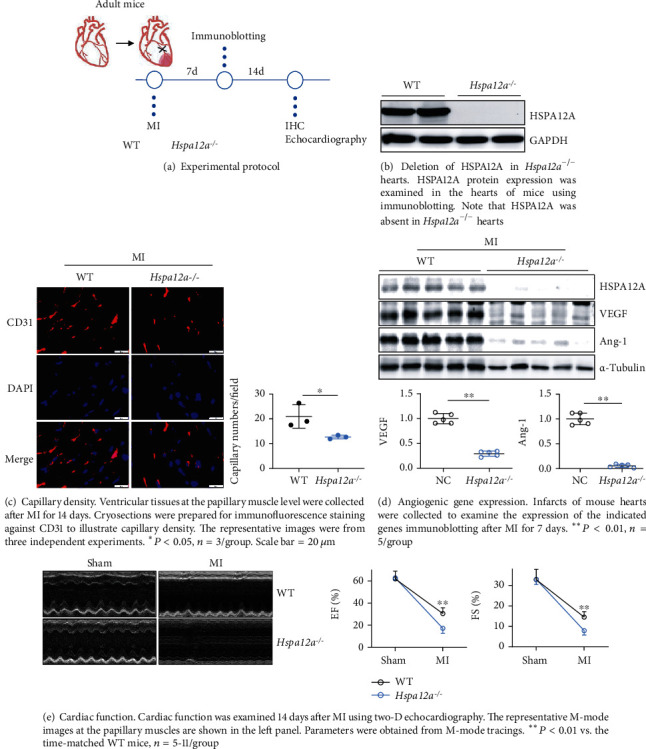
Deficiency of HSPA12A impaired angiogenesis and worsened cardiac dysfunction after MI in mice.

Finally, we were interested to know the biological roles of HSPA12A in post-MI angiogenesis and cardiac performance in intact animals. To this aim, mice with the HSPA12A knockout (*Hspa12a*^−/−^) and their gender-matched wild-type (WT) littermates were subjected to MI or sham surgeries (Figure 11(a)). The successful deletion of HSPA12A expression in *Hspa12a*^−/−^ hearts is shown in Figure 11(b). Angiogenesis that was examined by immunostaining for CD31 (PECAM-1) demonstrated that though no significant difference in capillary density was found between genotypes at basal levels (Figure S5), a lower capillary density was found in infarct regions of *Hspa12a*^−/−^ mice than in WT controls after MI for 14 days (Figure 11(c)). In supporting this, the hearts of *Hspa12a*^−/−^ mice displayed lower expression levels of VEGF and Ang-1 than the hearts of WT mice following MI (Figure 11(d)). In line with the reduced capillary density, *Hspa12a*^−/−^ mice demonstrated worsened cardiac dysfunction as indicated by lower left ventricular ejection fraction (EF%) and fraction shortening (FS%) than WT mice after MI for 14 days (Figure 11(e)). Additionally, histological examination by HE staining on paraffin-embedded sections, which were cut at left ventricular papillary muscle levels, showed a thinner free wall of the left ventricle in *Hspa12a*^−/−^ mice than WT controls after MI for 14 days (Figure S6). Altogether, deficiency of HSPA12A in mice impaired post-MI angiogenesis and cardiac function.

## 4. Discussion

The major finding of this study is that HSPA12A was upregulated during endothelial tube formation, and overexpression of HSPA12A increased proliferation, migration, and tube formation in endothelial cells. By contrast, the knockout of HSPA12A impaired angiogenesis and worsened cardiac dysfunction post-MI in mice. Further analysis revealed that HSPA12A promoted angiogenesis through activating AP-1 in p38- and ERK-dependent mechanisms. The findings identified HSPA12A as a novel angiogenic regulator and suggest that HSPA12A might serve as an alternative approach for the management of angiogenesis-related diseases such as myocardial infarction.

Heat shock proteins (HSPs) were originally identified as stress-responsive proteins required for cell survival during thermal stress, acting as molecular chaperones [39]. Now, it is clear that HSPs can respond to a wider variety of insults such as oxidants, heavy metals, and hypoxia/ischemia. According to their molecular size, HSPs are currently classified into structurally unrelated subfamilies, including HSPA/HSP70, HSPB/HSP27, HSPC/HSP90, HSPH/HSP110, and NDAJ/HSP40 [28, 40]. Several HSPs, especially HSP90, HSP70-1A, and HSP27, have been reported to stimulate angiogenesis to promote tumor growth [41, 42]. In hearts, HSP20 attenuates diabetic cardiomyopathy through improving angiogenesis in mice [43]. Intriguingly, we have demonstrated that HSPB1/HSP27 and HSPA12B are required for angiogenesis and cardiac functional recovery after MI in mice [2, 3]. In this study, we found that deficiency of HSPA12A in mice worsened cardiac dysfunction post-MI, and consistently, impaired post-MI angiogenesis was also detected in mouse hearts. The findings indicate that activating HSPA12A-dependent signaling might be beneficial for post-MI cardiac functional recovery by improving angiogenesis.

Angiogenesis could be either a physiological or a pathological process that forms new blood vessels trying to efficiently supply oxygen and nutrients during growth and development, as well as during wound healing after MI. Pathological angiogenesis is also essential for tumor progression and a variety of angiogenesis-related diseases (e.g., wet age-related macular degeneration, glaucoma, and diabetic retinopathy). Modulation of angiogenesis, therefore, is a promising therapeutic approach for related diseases. VEGF and Ang-1 have been regarded as crucial players in angiogenesis over several decades. Therapeutics targeting VEGF or Ang have been found to be clinically or preclinically useful for the suppression of pathological angiogenesis; however, two issues urgently need to be solved. First, there is still a lack of an ideal approach to promoting angiogenesis for improving post-MI wound healing; second, drug resistance is a current limitation of anti-VEGF therapy in clinical cancer treatment [6–11]. Therefore, identifying novel angiogenic factors remains a challenge for medical research. In this study, we found that HSPA12A, an atypical and distant member of the HSP70 family, showed upregulation during endothelial tube formation. Intriguingly, overexpression of HSPA12A promoted proliferation, migration, and tube formation of endothelial cells. By contrast, deficiency of HSPA12A impaired angiogenesis post-MI in mice. In supporting these results, HSPA12A displayed a positive regulation in angiogenic gene expression (VEGF, VEGFR2, and Ang-1). Altogether, our findings identified HSPA12A as a novel angiogenic activator.

AP-1 is a transcription factor regulating a variety of cellular processes such as proliferation, differentiation, and apoptosis. Recently, the emerging role of AP-1 has been demonstrated in angiogenesis and vascular development [13]. AP-1 is a dimer consisting of Jun (c-Jun, JunB, and JunD), ATF, or Fos. The transcription activity of AP-1 can be regulated by phosphorylation. For example, AP-1 can be phosphorylated by MAPKs and Akt, and the phosphorylated AP-1 will translocate to nuclei to drive the expression of VEGF and the VEGF downstream genes for angiogenesis [13–15, 38]. Conversely, VEGF can stimulate ERK activation [44]. Indeed, we found that overexpression of HSPA12A increases phosphorylation levels and nuclear localization of AP-1 in endothelial cells. We also found that HSPA12A activated ERKs and p38, whereas inhibition of ERKs and p38 diminished the HSPA12A-induced AP-1 phosphorylation, VEGF and VEGFR2 expression, and migration and tube formation of endothelial cells. The findings suggest that HSPA12A promoted angiogenesis through activating AP-1 in the p38- and ERK-dependent manners.

## 5. Conclusion

**Figure 12:**
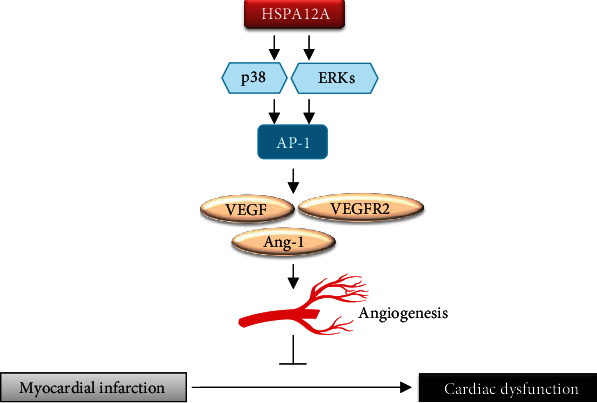
Mechanistic scheme. HSPA12A activates p38 and ERKs，by which to increase AP-1 phosphorylation and nuclear translocation to promote angiogenic gene expression and angiogenesis, and ultimately leads to improvement of myocardial infarction repairment. Overexpression of HSPA12A provides a therapeutic potential for the management of patients with myocardial infarction through promoting angiogenesis.

In summary, this study demonstrated that HSPA12A promotes *in vitro* angiogenesis and is required for myocardial angiogenesis post-MI in mice. This action was dependent, at least in part, on p38/ERK-mediated AP-1 signaling activation (Figure 12). The data suggest that HSPA12A expression may provide an effective strategy for post-MI cardiac functional recovery through promoting myocardial angiogenesis.

## Data Availability

The data used to support the findings of this study are available from the corresponding author upon request.

## Supplementary Materials

Supplementary 1 Figure S1: scheme of HSPA12A-adenovirus construction. Full length of mouse *Hspa12a* CDS was inserted in the multiple clonal sites (MCS).

Supplementary 2 Figure S2: overexpression of HSPA12A in HUVECs. Following infection with HSPA12A-adenovirus (*Hspa12a*^*o*/*e*^) or empty adenovirus (NC) for 24 h, HSPA12A expression was evaluated by immunostaining for HSPA12A. DAPI was used to counterstain nuclei. Scale bar = 20 *μ*m. *n* = 3/group.

Supplementary 3 Figure S3: overexpression of HSPA12A in HPAEC promoted migration capacity. Following infection with HSPA12A-adenovirus (*Hspa12a*^*o*/*e*^) or empty adenovirus (NC), a wound was made in the HPAEC monolayer. The healing of wounding was observed and expressed as the percentage of the original wounding area. ^∗∗^*P* < 0.01 vs. the time-matched NC. *n* = 3/group. Scale bar = 500 *μ*m.

Supplementary 4 Figure S4: effect of HSPA12A on c-Jun nuclear localization. Following infection with HSPA12A-adenovirus (*Hspa12a*^*o*/*e*^) or empty adenovirus (NC) in HUVECs for 24 h, immunostaining for HSPA12A and c-Jun was performed. DAPI was used to counterstain nuclei. Yellow arrows indicate that the HUVECs with stronger HSPA12A staining also contained stronger c-Jun staining in nuclei, whereas white arrows indicate the opposite effects. Scale bar = 20 *μ*m. *n* = 3/group.

Supplementary 5 Figure S5: effect of HSPA12A on myocardial capillaries at basal levels. Cardiac tissues without infarction were collected at papillary muscle levels of mice. After cryosectioning was prepared, immunostaining for CD31 was performed to indicate capillaries. DAPI was used to counterstain nuclei. Scale bar = 20 *μ*m. *n* = 3/group. WT: wild-type mice; *Hspa12a*^−/−^: HSPA12A knockout mice.

Supplementary 6 Figure S6: histological examination. Cardiac tissues were collected at papillary muscle levels after myocardial infarction for 14 days. After paraffin-embedded sectioning was prepared, hematoxylin-eosin (HE) staining was performed. Scale bar = 1000 *μ*m. *n* = 4/group. WT: wild-type mice; *Hspa12a*^−/−^: HSPA12A knockout mice.
