# Supplementary figures and images for: HSPA12A Stimulates p38/ERK-AP-1 Signaling to Promote Angiogenesis and Is Required for Functional Recovery Postmyocardial Infarction
Source: Oxid Med Cell Longev. 2022 Jun 22;2022:2333848. doi: 10.1155/2022/2333848 (PMC9247843; doi:10.1155/2022/2333848)

## Slide 1
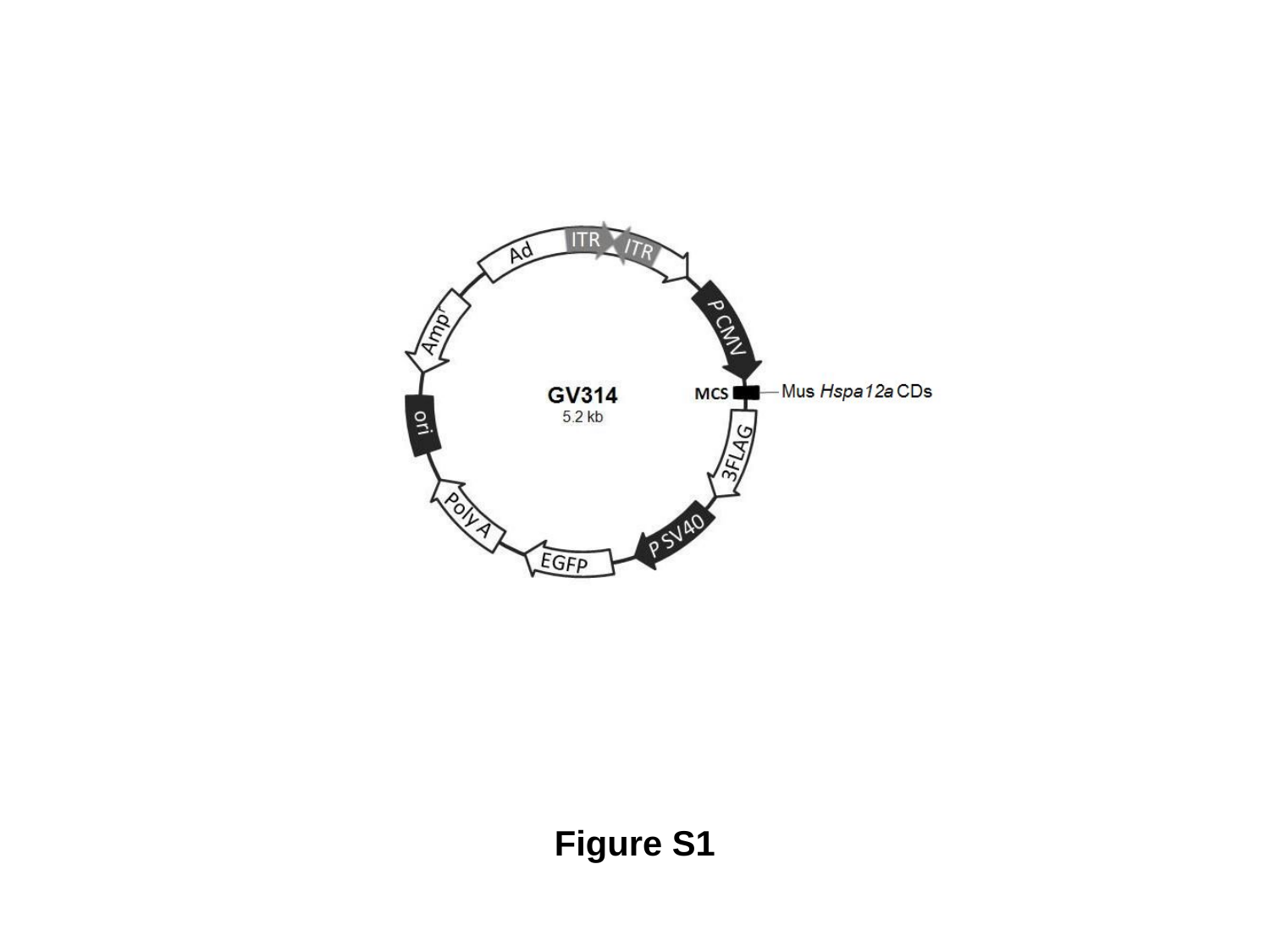

Figure S1

Supplement: Supplementary 1 — Figure S1: scheme of HSPA12A-adenovirus construction. Full length of mouse Hspa12a CDS was inserted in the multiple clonal sites (MCS). [file 2333848.f1.pptx]

## Slide 1
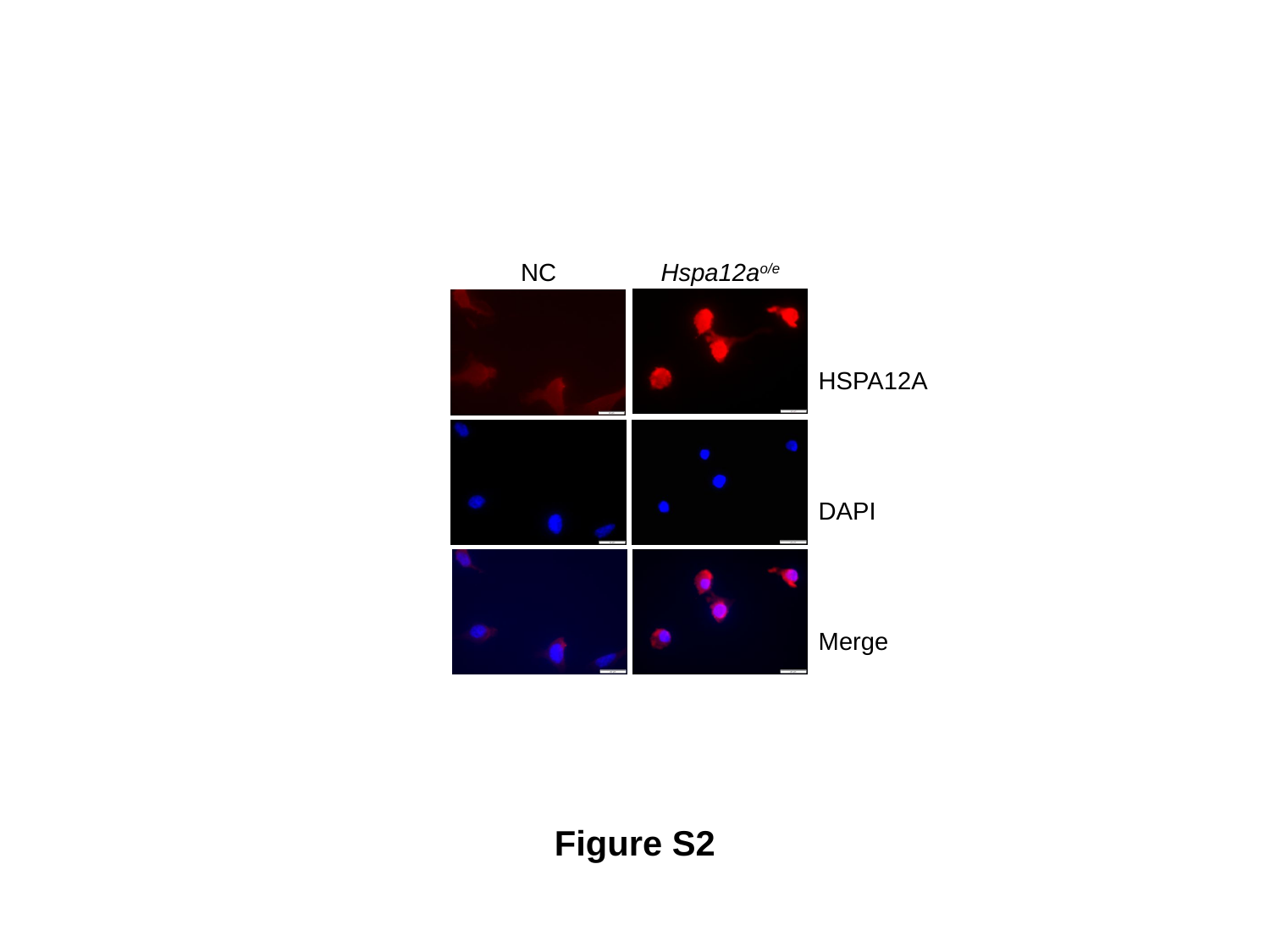

NC Hspa12ao/e
HSPA12A
DAPI
Merge
Figure S2

Supplement: Supplementary 2 — Figure S2: overexpression of HSPA12A in HUVECs. Following infection with HSPA12A-adenovirus (Hspa12ao/e) or empty adenovirus (NC) for 24 h, HSPA12A expression was evaluated by immunostaining for HSPA12A. DAPI was used to counterstain nuclei. Scale bar = 20 μm. n = 3/group. [file 2333848.f2.pptx]

## Slide 1
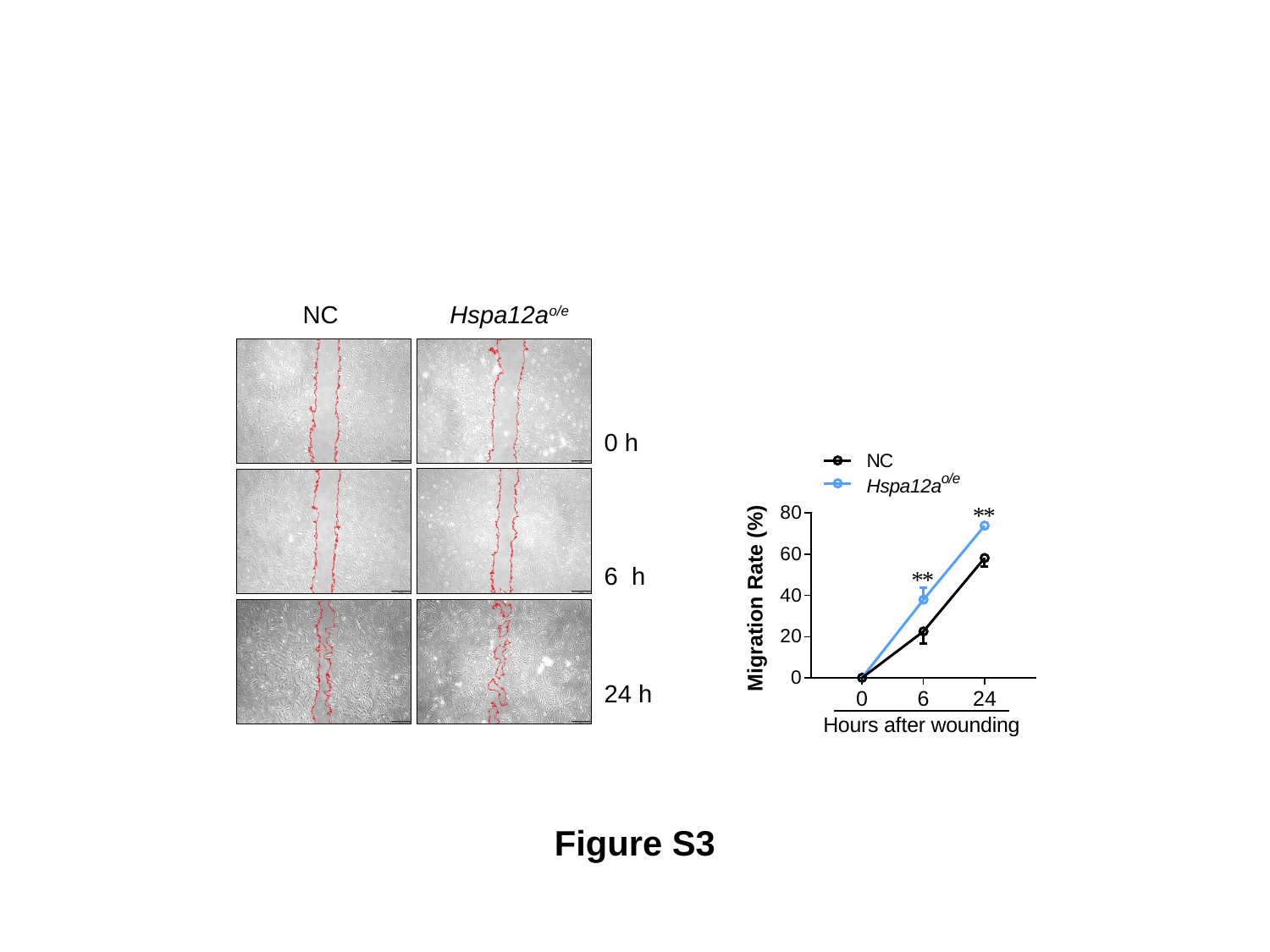

NC Hspa12ao/e
0 h
6 h
24 h
Figure S3

Supplement: Supplementary 3 — Figure S3: overexpression of HSPA12A in HPAEC promoted migration capacity. Following infection with HSPA12A-adenovirus (Hspa12ao/e) or empty adenovirus (NC), a wound was made in the HPAEC monolayer. The healing of wounding was observed and expressed as the percentage of the original wounding area. ∗∗P < 0.01 vs. the time-matched NC. n = 3/group. Scale bar = 500 μm. [file 2333848.f3.pptx]

## Slide 1
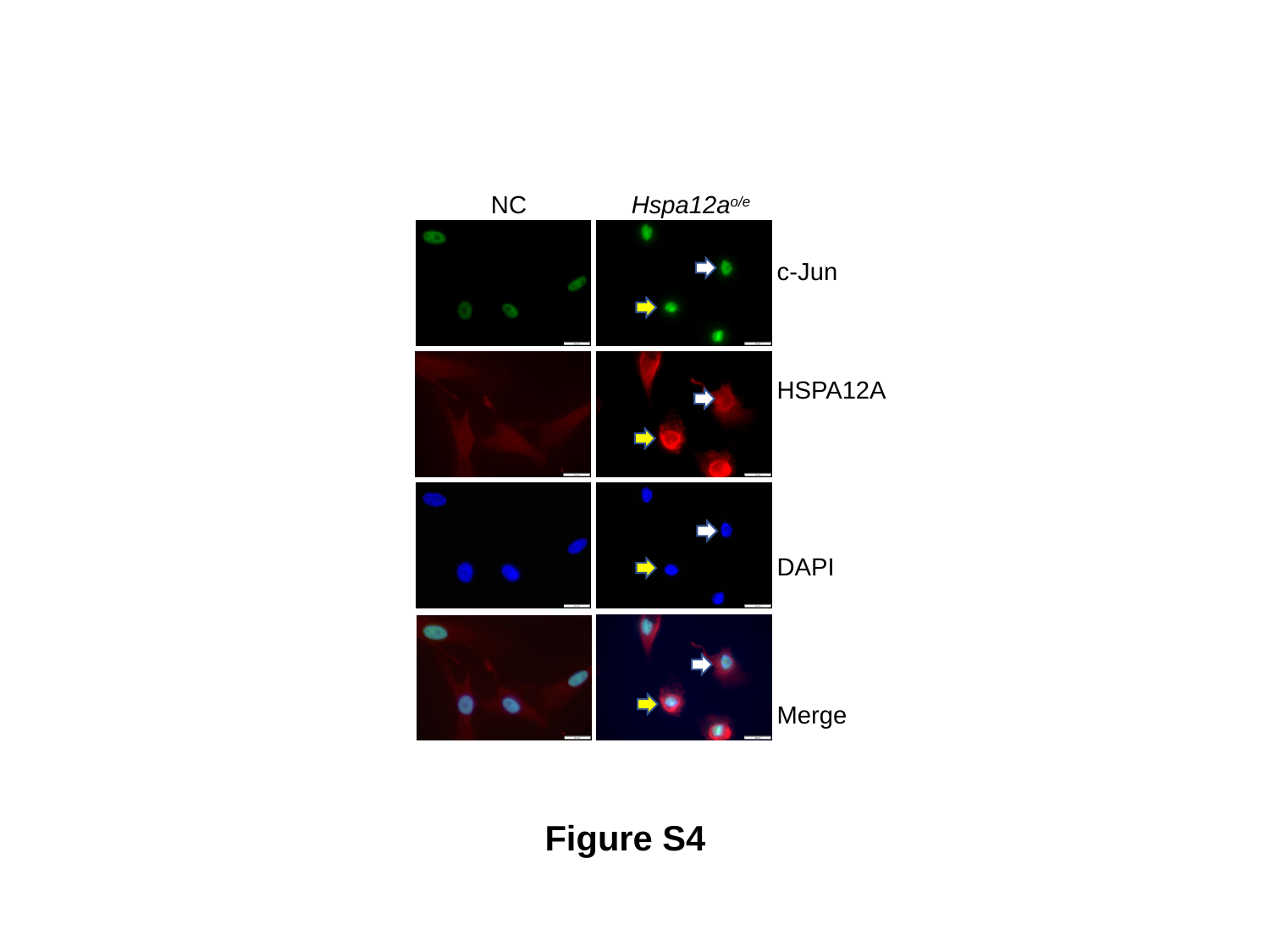

NC Hspa12ao/e
c-Jun
HSPA12A
DAPI
Merge
Figure S4

Supplement: Supplementary 4 — Figure S4: effect of HSPA12A on c-Jun nuclear localization. Following infection with HSPA12A-adenovirus (Hspa12ao/e) or empty adenovirus (NC) in HUVECs for 24 h, immunostaining for HSPA12A and c-Jun was performed. DAPI was used to counterstain nuclei. Yellow arrows indicate that the HUVECs with stronger HSPA12A staining also contained stronger c-Jun staining in nuclei, whereas white arrows indicate the opposite effects. Scale bar = 20 μm. n = 3/group. [file 2333848.f4.pptx]

## Slide 1
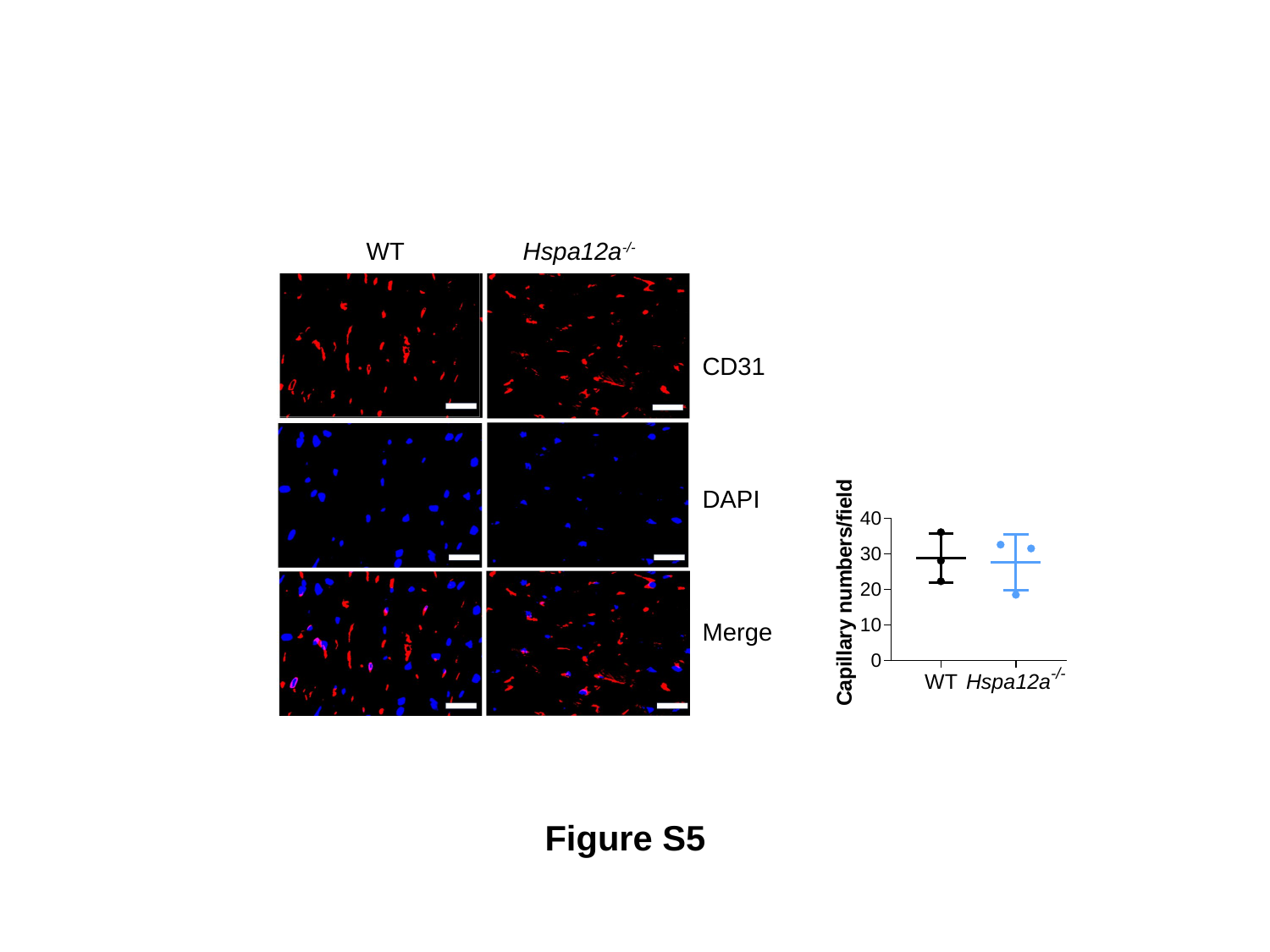

WT Hspa12a-/-
CD31
DAPI
Merge
Figure S5

Supplement: Supplementary 5 — Figure S5: effect of HSPA12A on myocardial capillaries at basal levels. Cardiac tissues without infarction were collected at papillary muscle levels of mice. After cryosectioning was prepared, immunostaining for CD31 was performed to indicate capillaries. DAPI was used to counterstain nuclei. Scale bar = 20 μm. n = 3/group. WT: wild-type mice; Hspa12a−/−: HSPA12A knockout mice. [file 2333848.f5.pptx]

## Slide 1
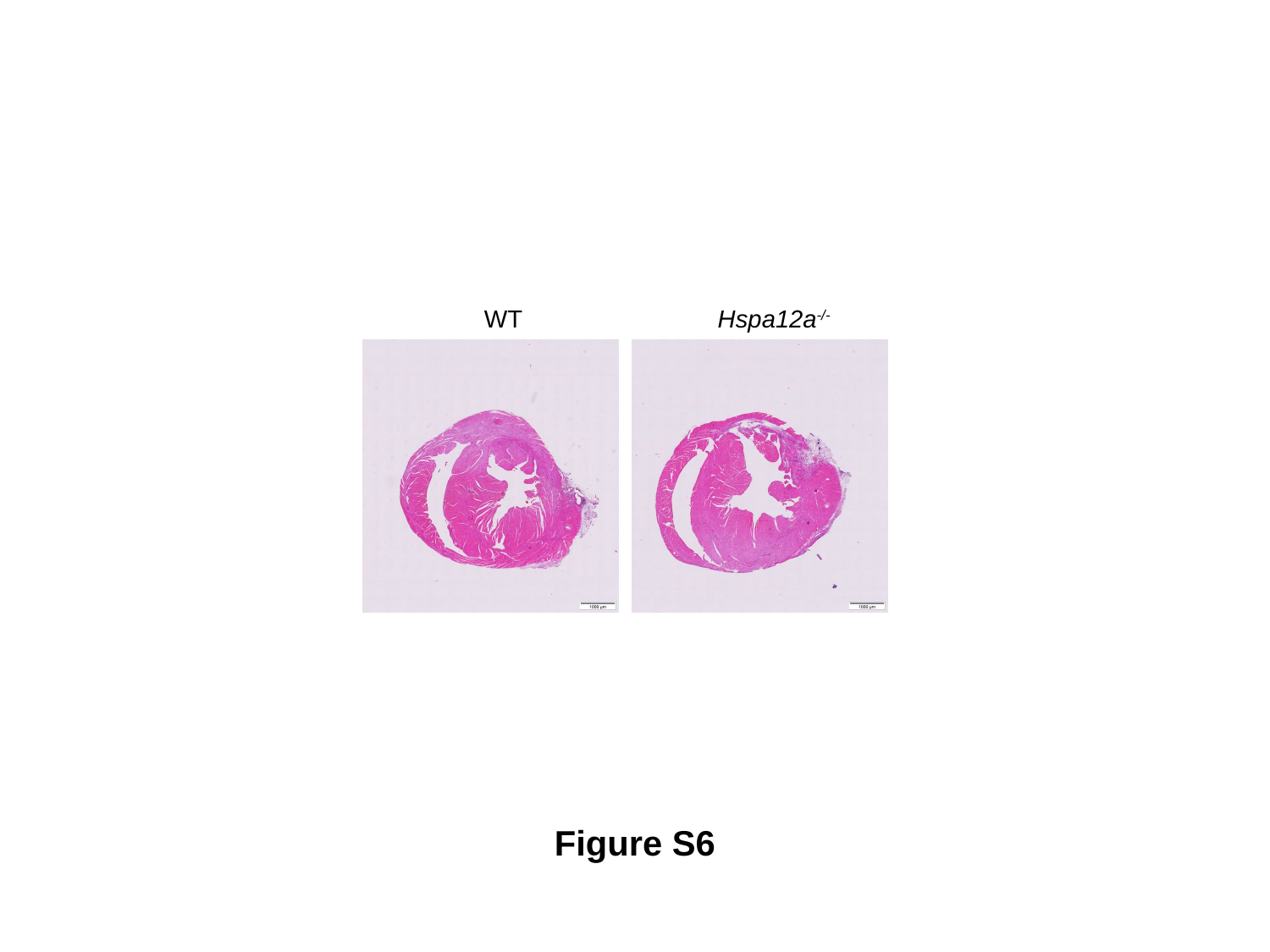

WT Hspa12a-/-
Figure S6

Supplement: Supplementary 6 — Figure S6: histological examination. Cardiac tissues were collected at papillary muscle levels after myocardial infarction for 14 days. After paraffin-embedded sectioning was prepared, hematoxylin-eosin (HE) staining was performed. Scale bar = 1000 μm. n = 4/group. WT: wild-type mice; Hspa12a−/−: HSPA12A knockout mice. [file 2333848.f6.pptx]
